# An Affordable NIR Spectroscopic System for Fraud Detection in Olive Oil

**DOI:** 10.3390/s23031728

**Published:** 2023-02-03

**Authors:** Candela Melendreras, Ana Soldado, José M. Costa-Fernández, Alberto López, Marta Valledor, Juan Carlos Campo, Francisco Ferrero

**Affiliations:** 1 Department of Physical and Analytical Chemistry, University of Oviedo, 33006 Oviedo, Spain; 2 Department of Electrical Engineering, University of Oviedo, 33204 Gijón, Spain

**Keywords:** instrumentation, Digital Light Processing (DLP), Digital Micromirror Device (DMD), Near-Infrared Spectroscopy (NIRS), olive oil, Partial Least Squares (PLS), Principal Component Analysis (PCA), spectroscopic

## Abstract

Adulterations of olive oil are performed by adding seed oils to this high-quality product, which are cheaper than olive oils. Food safety controls have been established by the European Union to avoid these episodes. Most of these methodologies require expensive equipment, time-consuming procedures, and expert personnel to execute. Near-infrared spectroscopy (NIRS) technology has many applications in the food processing industry. It analyzes food safety and quality parameters along the food chain. Using principal component analysis (PCA), the differences and similarities between olive oil and seed oils (sesame, sunflower, and flax oil) have been evaluated. To quantify the percentage of adulterated seed oil in olive oils, partial least squares (PLS) have been employed. A total of 96 samples of olive oil adulterated with seed oils were prepared. These samples were used to build a spectra library covering various mixtures containing seed oils and olive oil contents. Eighteen chemometric models were developed by combining the first and second derivatives with Standard Normal Variable (SNV) for scatter correction to classify and quantify seed oil adulteration and percentage. The results obtained for all seed oils show excellent coefficients of determination for calibration higher than 0.80. Because the instrumental aspects are not generally sufficiently addressed in the articles, we include a specific section on some key aspects of developing a high-performance and cost-effective NIR spectroscopy solution for fraud detection in olive oil. First, spectroscopy architectures are introduced, especially the Texas Instruments Digital Light Processing (DLP) technology for spectroscopy that has been used in this work. These results demonstrate that the portable prototype can be used as an effective tool to detect food fraud in liquid samples.

## 1. Introduction

Economically motivated food adulteration, also known as food fraud, is carried out to minimize production prices and increase benefits. The adulterated product loses quality or may even become harmful. Adulteration with lower costs alternatives is common in high-demand products such as milk and in products of high economic value whose price has been increasing in recent years, such as olive oil.

The limited production and high cost of the highly desirable olive oil make the olive oil susceptible to deliberated adulteration with low-cost and low-quality vegetable oils. The fat contained in such low-cost vegetable oils allows for conventional results to be performed to obtain satisfactory saponification values or refractive indexes, among others. This adulteration of olive oils with low-quality vegetable oils is a commercial fraud that can be focused on from two different points of view due to the quality of the product and a health and safety problem that is increased when the olive oil is consumed because of its nutritional benefits. Moreover, there are registered food safety occurrences in which oil adulteration was a major public health problem, such as in Spain in 1981. That year in Spain, several people died, and others suffered illnesses due to poisoning with adulterated oil [1,2]. The development of models for the authenticity of oils is thus of particular interest from an economic perspective and safety reasons.

The European Union has established food safety controls to avoid these episodes. Mostly, they are laboratory methodologies requiring expensive instruments, time-consuming and expert personnel to be carried out. Moreover, conventional methods cannot measure oil quality and safety analyses during certain processes, such as extraction or pressing. Some examples are high-performance liquid chromatography (HPLC) or gas chromatography (GC) with different detectors depending on the target analyte [3,4]. Therefore, a non-invasive, fast, or, if possible, real-time detection and affordable technique is required to determine the quality and safety parameters of the oil. Near-Infrared Spectroscopy (NIRS) technology has many applications in the food industry sector; it is used to analyze food safety and quality parameters along the food chain. Many studies have developed models that make it possible to discriminate between different types of oils or even between pure and adulterated oils. These studies allow us to establish a correlation between NIR profiles and safety parameters in oils, i.e., this technology can be applied for the detection of adulterations in oils [5,6,7,8].

Nevertheless, controlling impurity is a challenge involving authorities and the olive oil industry, requiring analytical alternatives based on real-time analysis that can be carried out onsite by non-expert personnel. An analytical alternative is those strategies based on portable instrumentation, such as NIR procedures. However, this standard instrumentation sometimes does not have the required instrumental characteristics to successfully solve this trouble, which is necessary for developing new portable instrumentation adapted to olive oil analysis. In this research, we focused our work on the development of a NIR prototype, a portable sensor, cheap, easy to use and adapted to olive oils analysis. To test our instrumental proposal, we have developed qualitative and quantitative chemometric models to detect adulteration of olive oils with low-quality vegetable oils.

The development of portable, small NIR spectrometers has advanced significantly over the past few years. These units operate using different optical diffraction grating properties, such as Fourier transform (Si-Ware NeoSpectra), linear variable filter (Viavi microNIR), and diffraction (Texas Instruments NIRscan Nano). Pasquini reviewed such instruments in a comprehensive review [9]. An extensive review by Crocombe [10] outlined the technologies used in portable spectroscopy and discussed their applications. Yan and Siesler [11] demonstrated the use of these low-cost FT-NIR, linear variable filters (LVFs), and diffraction NIR systems for both classification and measurement. Wolfrum et al. [12] compared the performance of low-cost NIRS to a conventional laboratory spectrometer. In Section 3, spectroscopy architectures are introduced, especially the Texas Instruments Digital Light Processing (DLP) technology for spectroscopy that has been used in this work [13].

## 2. Materials and Methods

### 2.1. Oil Samples

**Figure 1:**
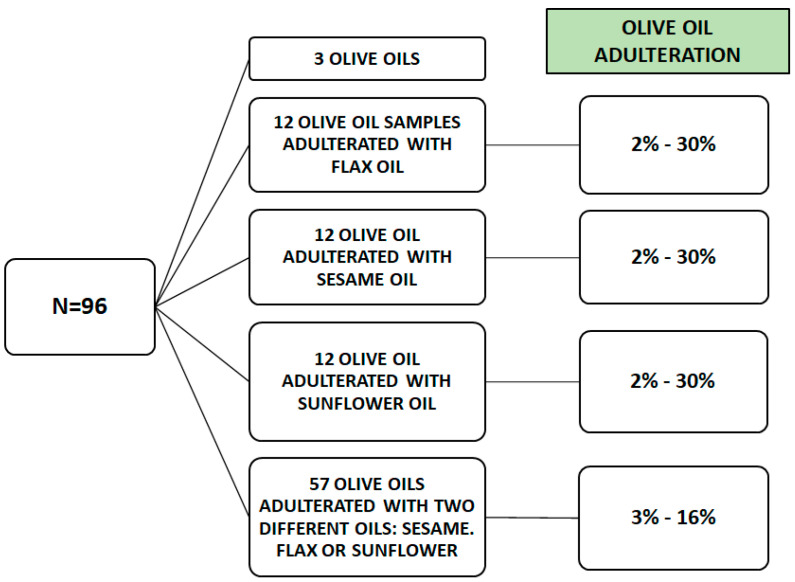
Sample preparation scheme (N = 96). Three pure olive oils, 12 samples of olive oil (4 samples of extra virgin olive oil, 4 samples of virgin olive oil and 4 samples of olive oil) adulterated with flax oil, 12 samples of olive oil adulterated with sesame oil, 12 samples of olive oil adulterated with sunflower oil, and 57 samples of olive oil adulterated with two of the selected adulterant oils.

This study used three different types of olive oil: extra virgin olive oil, virgin olive oil, and olive oil. Three varieties of seed oil adulterants, sesame oil, sunflower oil, and flax oil, were used for the mixture preparation. Figure 1 shows a scheme of samples employed in this study. As can be seen, three pure olive oils and 93 different binary and ternary mixtures of oil samples were made in this study.

As can be seen in Table 1, adulterated samples were prepared by adding uniformly different seed oils (flax, sesame and/or sunflower oil) to each olive oil. All the procedure was carried out as follows: each sample of olive oil was mixed with an adulterant oil varying the proportions of olive and adulterant oil, and the range of percentage of adulterant oil, in this case, is between 2 and 30% adulteration. Firstly, 36 binary samples were mixed. After that, 57 ternary mixtures were made by mixing each olive oil with two oils of the three possible adulterants in different proportions; the range of adulterant oils varied between 3 and 16%.

### 2.2. NIRS Analysis

**Figure 2:**
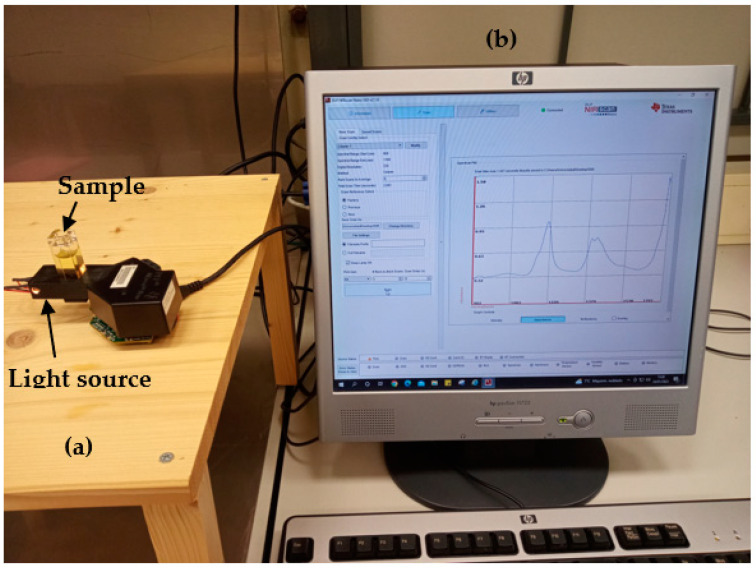
Transmittance measurement setup: (**a**) DLP NIRscan Nano EVM, (**b**) Graphical User Interface (GUI). Section 3.4 describes the GUI.

The oil mixtures were analyzed without pretreatment at room temperature (19 °C) in a standard quartz cuvette, 10 × 10 mm optical path, and a volume of 3500 mL (Hellma Analytics, Müllheim, Germany). The measurements were carried out in transmittance mode, as depicted in Figure 2. NIRscan is connected to the computer by a USB connection. The wavelength range was 901–1700 nm, and the path length was around 3 nm. A total of 228 points of different wavelengths were collected in each NIR analysis.

Before proceeding with the spectra collection, the instrumental conditions were optimized. The scanning mode Column or Hadamard and the number of scans to be averaged were evaluated and compared to select NIR experimental conditions. Each sample was divided into five portions, scanning each one separately. After optimizing experimental NIRS collection conditions, the final spectrum was the average of all the spectra of the same sample. In order to optimize the number of spectra to the average for each sub-sample, an olive oil sample was collected by averaging 1, 5, 10, 15, 20, 30, and 50 scans in both scanning modes. Once the data were collected, the values of these five different spectra’ root mean square error (RMS) statistic were calculated [14,15]. This statistic indicates the similarity between different spectra of the same sample analyzed in two different modes. The RMS value was used to select and compare repeatability and reproducibility conditions, allowing the selection of the scan model and the number of scans to be averaged [16,17]. The lower the RMS value, the more reproducible and repeatable the model. The value of the statistic for a sample is given by the following Equation (1):

(1) $$\;RMS=10^{6}\times \sqrt{\frac{\sum D^{2}}{n}};\;D=y_{a}-y_{b}$$

$y_{a}\;$= absorbance to λ for the average spectrum resulting from averaging several scans.

$y_{b}$ = absorbance to λ for the average spectrum resulting from averaging *b* number of scans.

n = number of spectral data.

### 2.3. Spectra Data Processing

The NIRS spectra collected with the prototype were transformed into a data matrix with X and Y variables defined as wavelength (X) and absorbance data (Y). Chemometric strategies were developed with Unscrambler X software (The Unscrambler X, CAMO Analytics AS, Oslo, Norway). Different strategies were developed to identify fraud in olive oil. One of them used categorical variables to identify the presence of seed oils in the olive oil samples, and the other one with the percentage of seed oil in the mixture to quantify the adulteration.

Principal component analysis (PCA) and partial least squares (PLS) were the chemometric approaches tested to attempt these qualitative and quantitative strategies. PCA was employed to detect potential spectral outliers and classify the adulteration type. PLS was the regression procedure employed to build the calibration models using the global spectrum (all the wavelengths 901–1700 nm) [18]. All the developed models were optimized using a random cross-validation method included in the software package, with 20 segments and five samples per segment. The optimal number of PLS factors was established considering the minimum residual variance.

Before PCA and PLS analysis, to minimize the light scattering phenomenon, the standard normal variation (SNV) mathematical treatment was applied to the spectra data matrix, on the raw data, and the data after applying different Savitzky–Golay (SG) derivative pre-treatments. To establish the successful model combination of pre-treatments (SNV, 1st and 2nd SG derivatives) were tested in this study. The pre-treatment code used in this study can be summarized using a four-digit notation (a b c d), where the first digit (a) refers to the order of magnitude of the Savitzky–Golay derivative (SG) (0 = underived spectrum, 1 = 1st derivative, 2 = 2nd derivative, etc.); the second digit (b) indicates the polynomial order of the derivate; and the third (c and d) digits indicate the size of the left and right intervals respectively, used for the derivative smoothing calculation. A total of 18 different calibration models (3 parameters × 6 chemometric strategies) were developed using different pre-treatments of the olive oils samples and PLS as the regression approach. The pre-treatments employed were as follows: SG 1 2 4 4, SG 2 2 4 4, SG 1 2 4 4 + SNV, SG 2 2 4 4 + SNV, SNV + 1 2 4 4, and SNV + 2 2 4 4. And the effect of applying scattering correction, SNV before and after derivation was also tested.

The best mathematical pre-treatments were selected based on the statistical criteria for each adulterant oil. This selection was based on the lowest calibration standard error (SEC) and cross-validation standard error (SECV), as well as the highest calibration determination coefficient (R^2^), cross-validation determination coefficient (r^2^) values, and Range error Ratio (SECV/Range). For the choice of the best-fitting equation, it was also considered that the values of the calibration determination coefficient and the cross-validation coefficient did not have values particularly different from each other [19,20].

## 3. Instrumentation

### 3.1. Spectroscopy Architectures

**Figure 3:**
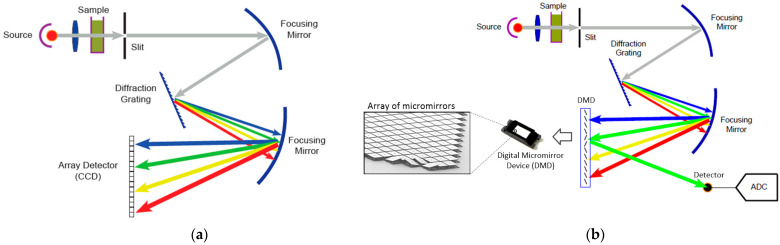
Spectroscopy architectures: (**a**) traditional (**b**) digital light processing (DLP).

Hand-held NIR spectrometers can also be classified based on the type of detector: array detectors and single-detector instruments. Figure 3a shows the traditional architecture of a spectrometer using an array detector [21]. Due to its high cost, large volume, and complicated operation, the conventional spectroscopy system is generally suitable for laboratory environments.

Although excellent results can be obtained with array detectors, to develop a low-cost, portable, and user-friendly spectral detection system, which can be adapted to the on-site scenario, we chose the DLP architecture from Texas Instruments, depicted in Figure 3b. The main difference is using a digital micromirror device (DMD) inserted into the optical path to select specific wavelength regions for measuring by a single detector. The selection of individual wavelengths is accomplished by selectively turning columns of mirrors on or off to reflect or transmit only the desired wavelengths to the detector. The NIR wavelength region allows using a high-performance, cost-effective single-element detector while providing wavelength selection agility, speed, and mechanical stability.

**Figure 4:**
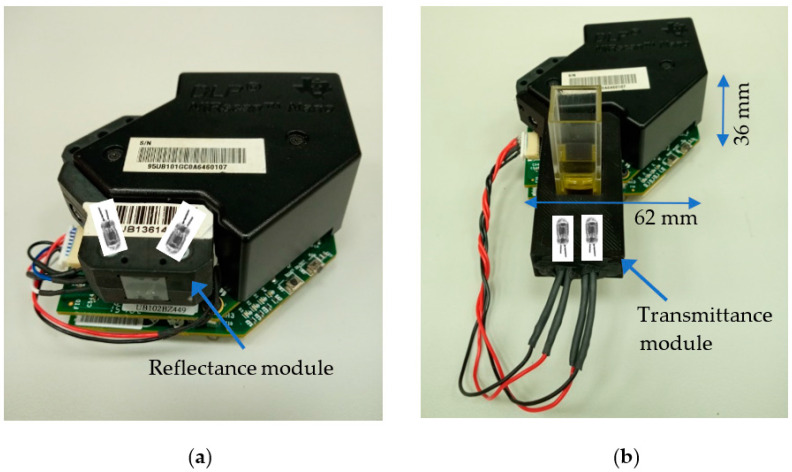
DLP NIRscan Nano EVM with (**a**) reflective module and (**b**) transmittance module.

The DLP NIRscan Nano spectrometer evaluation module (EVM, Texas Instruments Incorporated, Dallas, TX, USA, $999) is equipped in origin with a diffuse reflectance illumination module as shown in Figure 4a. To detect the presence of target compounds in olive oil, we designed a transmittance module shown in Figure 4b using a 3D printer.

### 3.2. Optical Considerations

**Figure 5:**
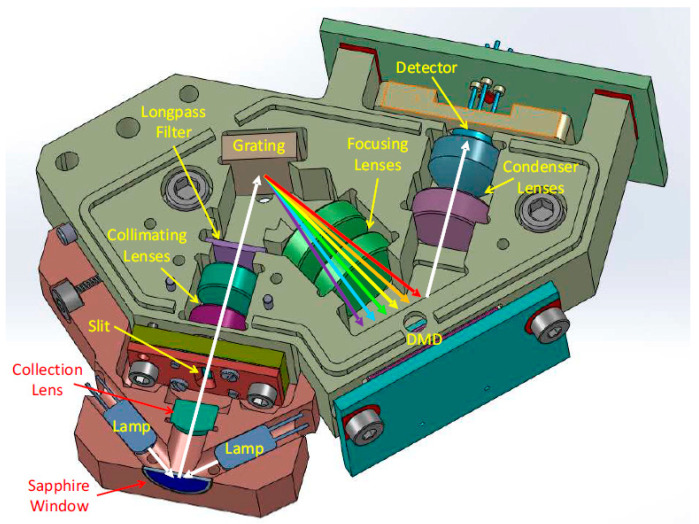
Interior view of the DLP NIRscan Nano-optical architecture.

Figure 5 displays the optical elements of the DLP NIRscan nano reflectance module [22]. It is mounted on the top of the electronics subsystem. The reflective mode collects light reflected by the sample and passes it through a slit. The slit width is chosen to balance wavelength resolution with a signal-to-noise ratio (SNR) depending on the desired specifications of the system. The DMD selects specific wavelengths and directs them to a single-point photodetector. The DMD accomplishes wavelength selection through a set of patterns applied to the micromirrors. The sequence of patterns forms a scan configuration. Thus, the slit width, DMD array, scan configuration, and DMD column width influence the resolution and maximum amount of light on the photodetector. Table 2 shows the main specifications of the NIRscan Nano EVM [22].

### 3.3. Hardware Considerations

**Figure 6:**
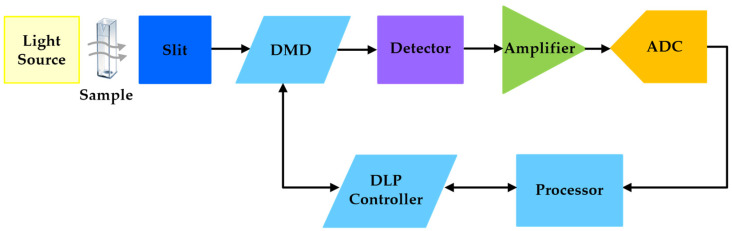
Basic block diagram of the DLP NIRscan Nano hardware.

Figure 6 shows a basic block diagram of the DLP spectrometer with the transmissive illumination module. An exhaustive description of these components can be found in [23].

**Figure 7:**
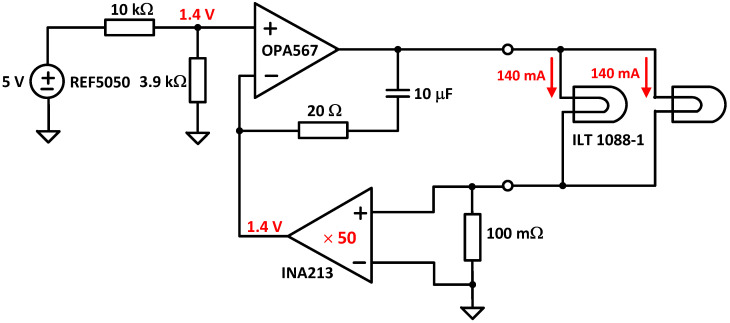
DLP NIRscan Nano lamp driver.

**Figure 8:**
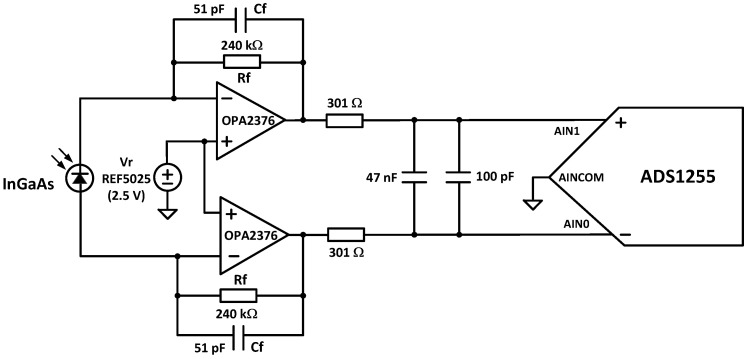
Transimpedance amplifier circuit.

- *Light source*: It consists of two lens-end Tungsten filaments. They are designated as lens-end lamps because the front end of the glass bulb is formed into a lens to direct more light from the filament to the sample test region. Tungsten halogen lamps are well suited as spectrometer light sources due to their broadband infrared radiation. The transmission module was equipped with two ILT 1088-1 lens-end lamps from International Light Technologies [24]. Figure 7 shows the lamp driver. It provides a constant current of 280 mA at 5 V. The light output of this lamp is sufficient for cuvettes with path lengths of 2–13 mm.
- *Slit*: The input slit specifications affect the ability to couple light into the spectrometer and its spectral resolution. The slit width should be chosen to create an image width at the DMD corresponding to a desired spectral resolution. In addition, the slit length should be large enough to illuminate the full extent of the DMD panel, maximizing the system’s light throughput. Narrowing the slit width increases the spectral resolution of the system but requires a higher-performance optical system to sharply image the smaller slit onto the DMD [25].
- *DMD array*: The size of this component determines the maximum light collection area and the resolution of the system. The resolution of the system depends on the wavelength spectrum that is spread across one dimension of the DMD (for example, width), the slit width, and the DMD pattern width. The other dimensions of the DMD (for example, height) and the optical transfer function of the system determine the amount of light collected.
- *Photodetector*: For NIRS between 900 and 2500 nm, an InGaAs photodiode is the preferred photodetector due to its high quantum efficiency and responsivity. Typically, the photodiode produces a very small current signal proportional to its photosensitive area. A large photosensitive area produces more current in response to light at the expense of higher terminal capacitance. The higher capacitance will result in a slower response to light or lower bandwidth. This capacitance will also affect the capacitive feedback compensation (Cf in Figure 8) of the transimpedance amplifier (TIA). The photodetector also has a dark current when no light is incident. Thus, photodiode selection is a trade-off between cost, wavelength range, photosensitivity, capacitance, and dark current. DLP NIRscan Nano uses the Hamamatsu InGaAs G12183-120K photodiode [26].
- *Amplifier*: Once the light is converted into a current at the photodetector, a TIA is used to convert the current signal into a voltage. The TIA architecture is best suited for photodiodes that produce higher input currents, achieve wide analog bandwidth, offer high flexibility with simple changes of feedback elements, and handle the high-speed conversion. For noise immunity, the TIA employs a differential signal, with a gain double the feedback resistance, Rf, in Figure 8.
- *Analog-to-Digital Converter (ADC)*: The ADC converts the voltage into a digital signal representation. For best results, an extremely low-noise, high linearity, high-resolution analog-to-digital converter minimizes the noise added by the conversion. To optimize the system’s digital resolution, the ADC’s dynamic range and reference voltages must be matched with the maximum photodetector signal. DLP NIRscan Nano uses the Texas Instruments 24-bit ADS1255.

### 3.4. Software Considerations

A key characteristic of this micro-spectrophotometer is that the relevant software can be downloaded from the manufacturer’s website, along with extensive hardware documentation and free access to routines for programming and communicating with it. It is essential for those who want to develop new applications for the instrument.

**Figure 9:**
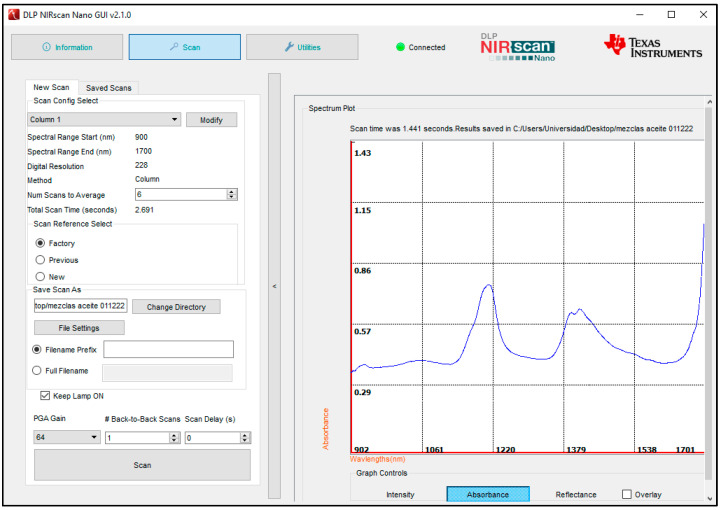
DLP NIRscan Nano GUI Scan Screen.

Figure 9 displays the spectrum plot and the controls for scan configurations and parameters of the DLP NIRscan Nano GUI [27]. To create a scan configuration, the first step is to enter the number of scans to average. Averaging each wavelength point across multiple scans reduces noise while increasing the total scan time.

The second step is to enter the number of sections of a scan. A scan can be broken up into 1–5 sections. Scans with the same width and resolution should be done in only one section. More than one section must be created to create a fast scan with less resolution on wavelengths with less information and a higher resolution on wavelengths with areas of interest. Each section can have an individual set of the following parameters:

- *Method*: This controls the scanning process. The DLP NIRscan Nano comes preloaded with two scan configurations from the factory: Column or Hadamard. The Column scan selects one wavelength at a time. The Hadamard scan creates a set with several wavelengths multiplexed at a time and then decodes the individual wavelengths. The Hadamard scan collects much lighter and offers a higher SNR than a column scan [28]. However, this is very dependent on the spectrum being measured and the system used to measure it. To identify adulterations in different olive oils, we consider the Column method to be more effective because the reproducibility study provided better results for the Column method, as stated in this work.
- *Wavelength range*: Start and End wavelengths (in nm) or spectral range of interest for the scan. The minimum wavelength is 901 nm, and the maximum wavelength is 1700 nm.
- *Width in nm*: This number selects the width of the groups of pixels in the generated Column or Hadamard patterns. The options displayed correspond to the width of the dispersed spectrum in nm across the quantized pixel width.
- *Digital Resolution*: This number defines how many wavelength points are captured across the defined spectral range. This corresponds to the number of patterns displayed on the DMD during the scan. By increasing the digital resolution, the spectrum is oversampled. In general, set this resolution to oversample at least twice the desired full-width half maximum desired.
- *Exposure Time*: For scan configurations with one section, the exposure time is set to 0.635 ms. For scan configurations with more than one section, the exposure time can be individually set for each section in the range of 0.635 to 60.960 ms.
- *Number of scans to average*: This determines how many times the pattern set for a scan will be cycled through on the DMD. The microcontroller collects and averages the data, which lowers noise as additional scans are averaged. For scans where noise is a problem, this can be increased.
- *Scan Reference Select*: This button allows the user to choose the reference for the absorbance or reflectance graph. For transmittance sampling, data are taken with no sample in the cuvette.

The distribution of SNR across the spectrum is critical (i.e., SNR at each wavelength), especially for a DMD-based spectrometer where uniform illumination across the mirror array is important. To test SNR at a particular scan setting, the following procedure was used [29]:

1. Execute scan k times (we used k = 4) with scan time t and no time between scans.
2. Compute the difference vector of the intensities at wavelength n.
3. Compute the average of the intensity measurements at wavelength n.
4. Compute the standard deviation of point 2.
5. Compute the SNR as the ratio between points 3 and 4.
6. Repeat points 1 to 5 at other wavelengths between 900 to 1700 nm.

**Figure 10:**
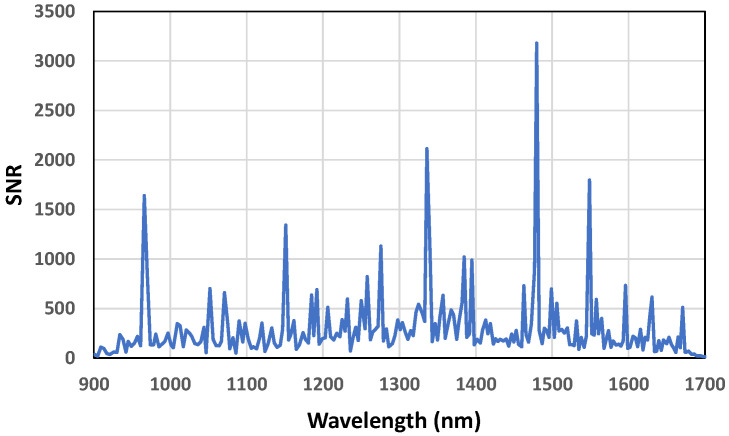
SNR of the Column scan as a function of wavelength.

Following the above procedure, Figure 10 shows the SNR of the Column scan as a function of the wavelength in DLP NIRscan EVM.

## 4. Results and Discussion

**Figure 11:**
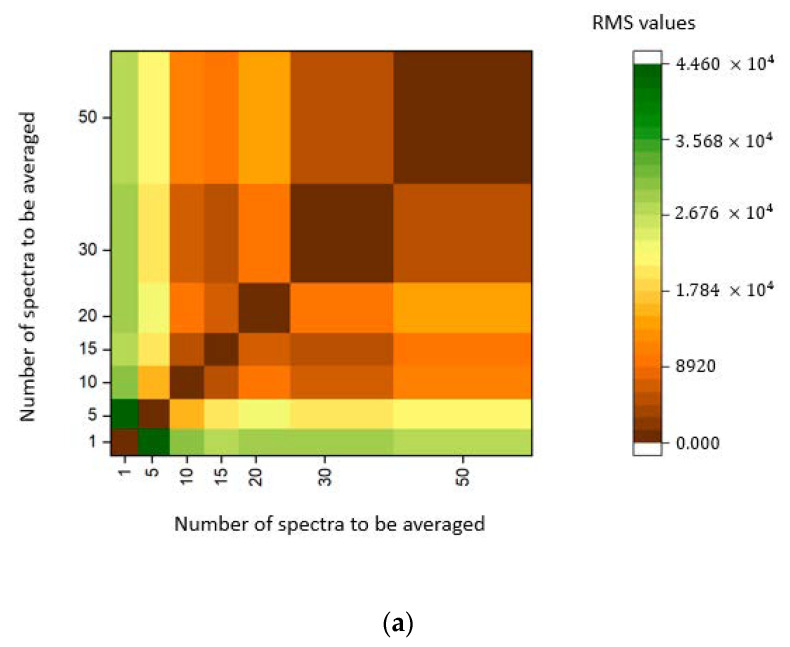

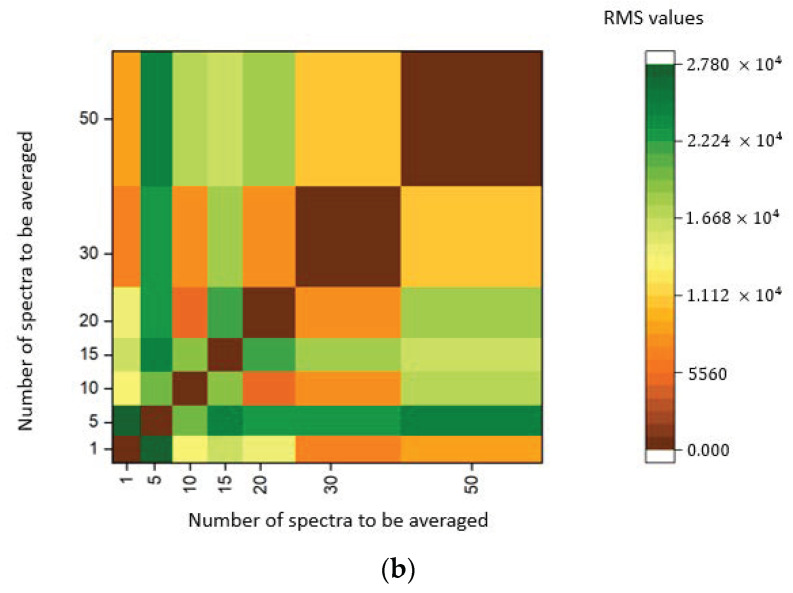
Heatmap showing the Root Mean Square (RMS) values for each number of scans to be averaged. (**a**) Heatmap with the RMS values using the Column scan model; (**b**) heatmap with the RMS values using the Hadamard scan model.

The success of a NIRS methodology depends on the quality of the collected spectral information. Taking into account this consideration, the first step carried out in this study was the selection of the instrumental conditions. The developed prototype allows collecting spectra using two different scan modes, Column and Hadamard. In both modes, the final spectrum can be the average of a limited number of individual spectra. As detailed in Section 2, the scanning mode and the number of spectra to average were optimized by calculating RMS as spectra reproducibility parameter. Figure 11 shows the obtained results combining scan mode and the number of spectra to average in a heatmap.

As can be seen in Figure 11, in comparing the Column and Hadamard heatmaps, a more homogeneous color (brown color) indicates lower RMS values and minor differences between spectra of the same sample collected in reproducibility conditions. The Column mode was selected as the scanning mode for further studies. Looking at the Column heat map, it is observed that, for the Column model, we see that even averaging 15 scans, the value of the RMS statistic is low (homogeneous brown color). To improve the spectra quality, minimize the analysis time, and be able to scan as many samples as possible, the number of spectra to average in each analysis was fixed in 30 scans per measurement of each spectrum.

**Figure 12:**
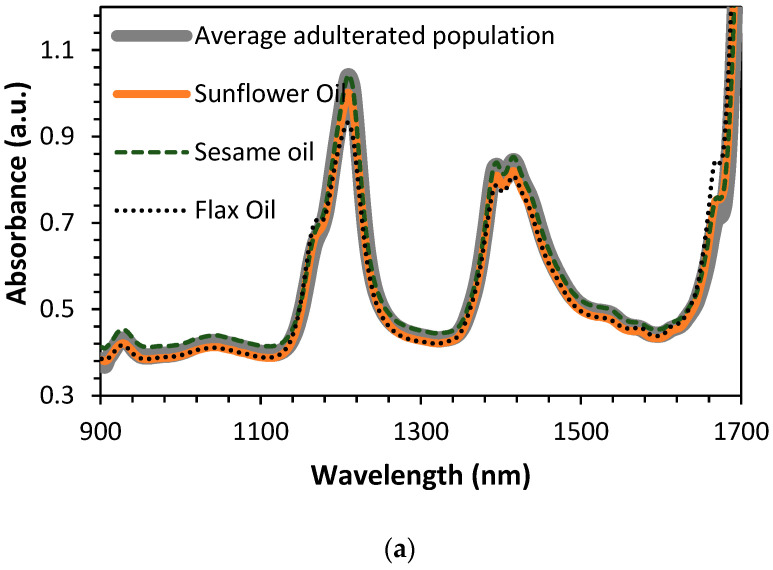

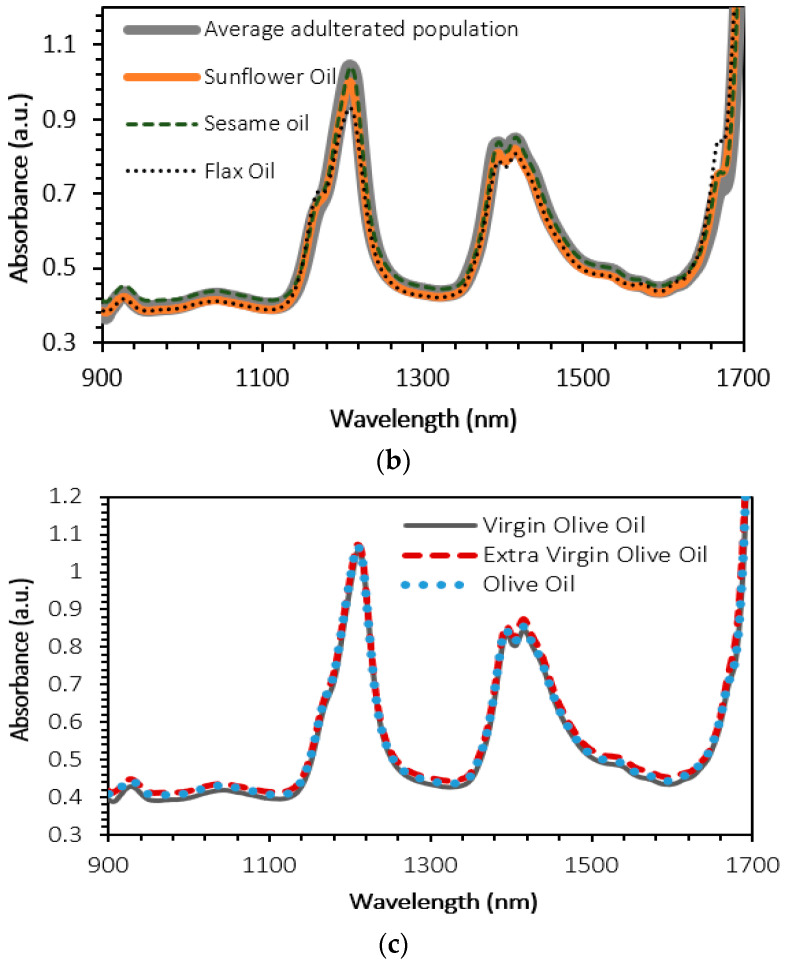
Raw spectra of all pure oils: (**a**) raw spectra of all mixtures, (**b**) seed oils and average spectrum of all adulterated samples, and (**c**) olive oils spectra.

All samples were scanned with the proposed prototype using previously detailed instrumental conditions. Figure 12 shows the raw spectra of pure olive samples, seed oils (sesame oil, sunflower oil, and flax oil), and the average spectra of all adulterated samples.

**Figure 13:**
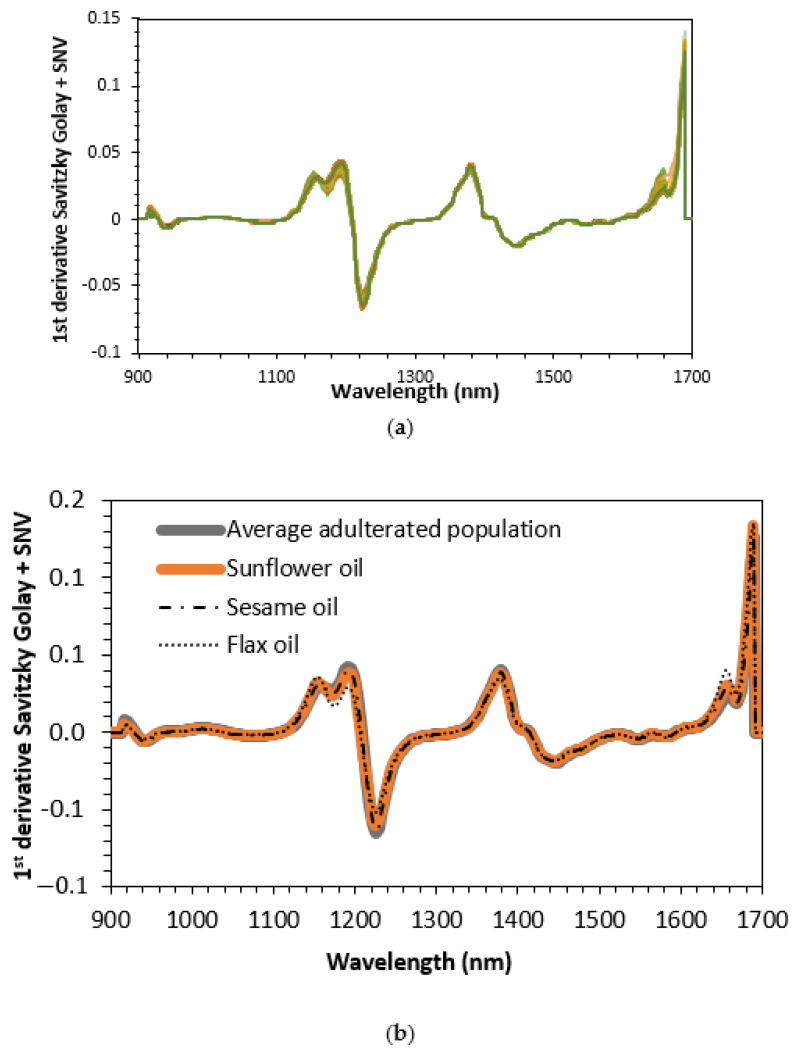

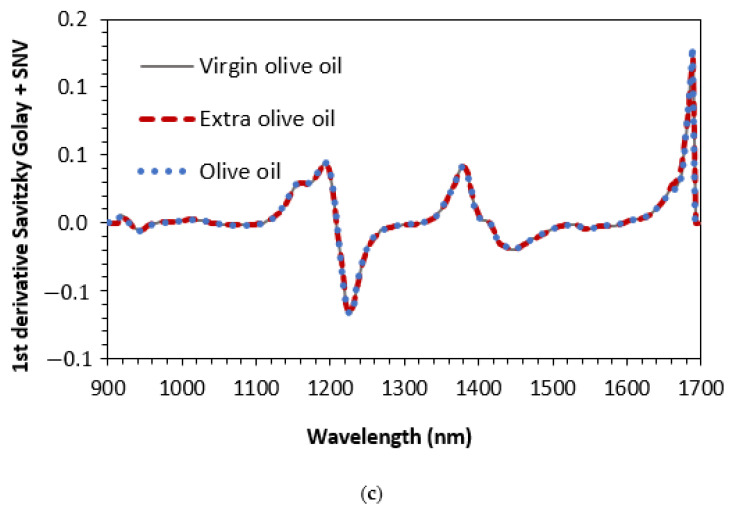
First-derivative Savitzky–Golay spectra plus Standard Normal Variate (SNV) of all pure oils: (**a**) all mixtures, (**b**) olive oils, and (**c**) seed oils and average spectrum of all adulterated samples.

In the NIR range of the prototype, there are the following characteristic bands of the oil’s spectra. The bands observed around 1200 related with C-H (CH_2_) second overtone vibration and broadband with a double peak between 1400–1500 nm due to O-H first overtone and C-H (CH_2_) combination [20,30]. In Figure 13, the 1st derivative plus SNV of all samples involved in this study are plotted, as well as the average spectra of all adulterated oils. As can be seen, comparing Figure 13a–c, some differences are observed at 1160 and 1660 nm. Olive oil spectra, for both wavelengths, show a small shoulder, whereas, in seed oils and adulterated samples, a clear peak appears at both wavelengths when plotting spectra data.

**Figure 14:**
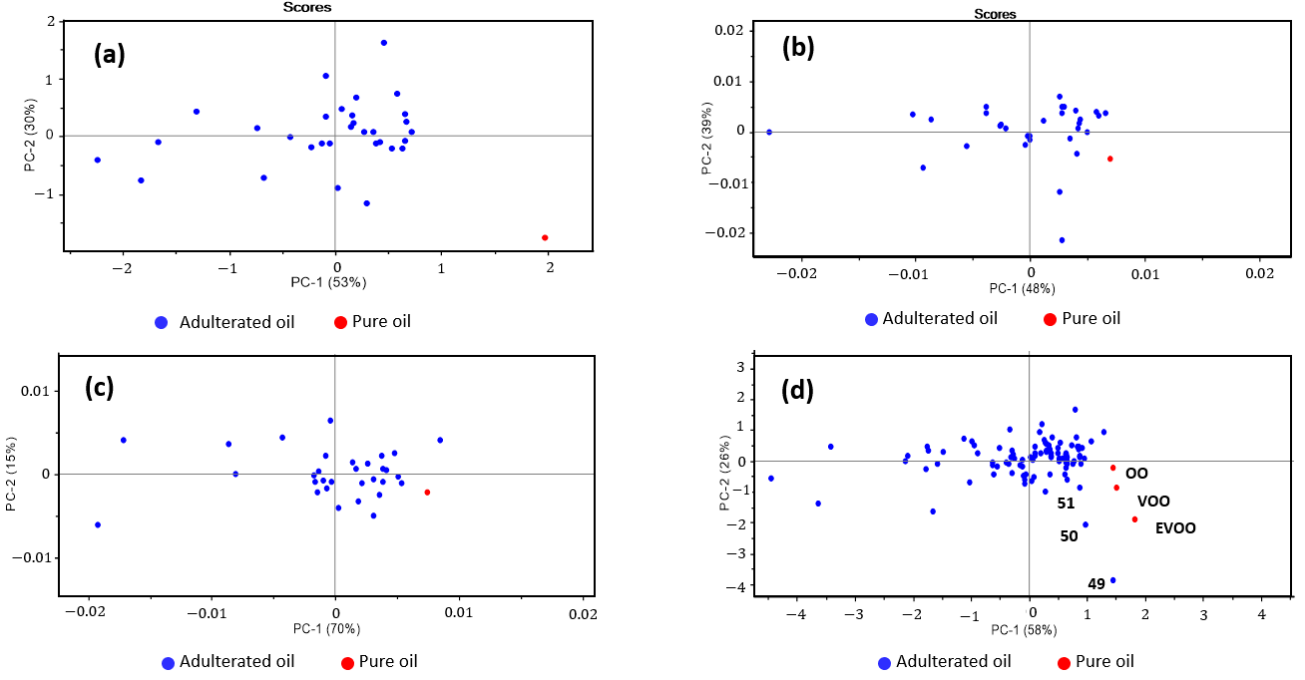
Principal component analysis of three olive oils and their corresponding mixtures: (**a**) extra virgin olive oil (EVOO) and mixtures of EVOO and other seed oils, (**b**) virgin olive oil (VOO) and mixtures of VOO and other seed oils, (**c**) olive oil (OO) and mixtures of OO and other seed oils, and (**b**,**d**) all the samples pure and adulterated in the sample set.

After plotting the spectra, principal component analysis (PCA) was carried out for each type of olive oil with its corresponding mixtures and all sample sets (N = 96) involved in this study. Different approaches were evaluated for PCA analysis using SG first and second derivatives. Figure 14 summarizes the best-obtained results.

As can be seen in Figure 14, comparing each olive oil with its mixtures, a clear difference is observed between adulterated and pure olive oils. For all the olive oils, a positive correlation is observed for PC1 and a negative for PC2. In Figure 14d, it is possible to identify the three pure olive oils separated from the rest of the samples. However, it was observed that the three samples presented a pattern significantly similar to the pure olive oil samples, as they are parallel points to the three pure samples. Samples 49, 50, and 51 were found to be three blends of virgin olive oil with two adulterant oils. In all cases, the mixture is composed of 90% virgin olive oil and 5% of one adulterant and 5% of another adulterant; in the case of sample 49, sesame and flax oil; in sample 50, sunflower and flax oil; and in 51, sesame and sunflower oil.

After evaluating the classification procedure, the next step was to perform the calibration models with all the spectra, using the PLS regression model and cross-validation with random groups. As indicated in Section 2, different mathematical pretreatments were tested before developing calibration models; 18 models with six mathematical pretreatments were evaluated for the calibration of three adulterant oils: sesame oil, sunflower oil, and flax oil. Table 3 summarizes all the results obtained for each treatment and type of adulteration.

As can be seen in Table 3, all coefficients of determination for calibration values (R^2^) are higher than 0.74 except for sunflower oil applying SG 2 2 4 4 plus SNV pre-treatment (0.313). The coefficient of determination for cross-validation values is similar; all values are greater than 0.6 except for sunflower oil with the SG 2 2 4 4 plus SNV pre-treatment, whose value is 0.276. The highest R^2^ value is obtained with the SG 1 2 4 4 plus SNV pre-treatment for sunflower oil, whose calibration error (SEC) is 2.256.

An overall view of the results shows that the R^2^ values for each oil are quite similar for sesame oil and flax oil (values between 0.740 and 0.830) by applying the different pretreatments. Still, the values of the coefficient of determination for the calibration of sunflower oil vary greatly depending on the pretreatment selected, ranging from the minimum value obtained 0.313 for SG 2 2 4 4 plus SNV to the maximum value 0.921 for the pretreatment SG 1 2 4 4 plus SNV. However, these marked differences, observed for R^2^, are not so evident for r^2^.

The best coefficients of determination for calibration were selected, evaluating comparatively both coefficients of determination for calibration and cross-validation. As seen in Table 3, there are no wide differences between one or other math pretreatment nor related to scatter correction (SNV) before or after derivative pretreatment. All the values for R^2^ ranged between 0.921 and 0.747, and the 1st derivative plus SNV as mathematical pre-treatment for adulteration with sunflower oil or with sesame oil, respectively. Values of coefficients of determination for cross-validation regression models (r^2^, see Table 3) ranged between 0.771 for adulteration with sesame oil (pretreatment of the 1st derivative) and 0.695 for adulteration with sunflower oil (pretreatment with SNV plus the 2nd derivative). Adulteration with sunflower oil, using pretreatment of 2nd derivative and, after that, SNV for scatter correction showed no satisfactory results for qualitative and quantitative considerations with values of 0.313 and 0.275 for R^2^ and r^2^, respectively.

Considering the calibration errors and the dimensionless statistic values, Range Error Ratio (RER = Range/SECV), as shown in Table 3, the best results were obtained for all adulterations with RER values of 7.1, 9.0, and 5.6 for sesame oil, flax oil, and sunflower oil respectively. All these models were developed after applying a 1st-derivative pretreatment to spectra data. In addition to that, for sunflower and flax adulteration, a SNV scatters correction prior to or after derivative pretreatment was required to improve statistics results.

Regarding standard error (SEC and SECV), it should be noted that the low range of the adulteration assayed (2%) is within the detection limit of the proposed method, being necessary to increase variability in the low range and perhaps to develop a specific model depending on the adulteration.

After obtaining and processing the spectra, the results shown in Table 3 must be simply presented to determine whether olive oil is adulterated. To implement this task, a microcontroller-based circuit should be designed and connected to the NIRscan Nano. The display can be two LEDs, for instance, a green LED in case there is no fraud and a red LED in case of fraud. This is the next step we are working on.

Table 4 shows different analytical techniques used to detect olive oil fraud. The discrimination or classification procedure and the most relevant advantages and inconveniences are included to provide all the information about detecting olive oil adulteration.

## 5. Conclusions

In this work, we presented and tested a portable and cost-efficient Near Infrared Spectroscopic prototype for fraud detection in olive oil. For this purpose, a transmittance module was developed for the Digital Light Processing NIRscan Nano EVM. Detection of fraud in liquid samples such as olive oils requires the collection of robust spectra with relevant information. All classification and calibration models showed satisfactory results. For all seed oils, the coefficient of calibration determination is greater than 0.80 and for cross-validation around or higher than 0.7. These results confirm the excellent characteristics of this portable prototype. It can be used as a tool to detect food fraud in liquid samples. We also included key instrumental considerations that should be considered in designing a hand-held Near Infrared Spectroscopic system.

## Figures and Tables

**Table 1:** Percentage distribution of binary (olive oil and one adulterant oil) and ternary (olive oil and two adulterant oils) mixtures.

Binary Mixtures		Ternary Mixtures		
% Olive Oil *	% Adulterant Oil	% Olive Oil *	% Adulterant Oil	% Adulterant Oil
98	2	90	5	5
90	10	80	10	10
80	20	70	15	15
70	30	88	9	3
		76	8	16
		92	5	3
		86	11	3
		82	10	8

Adulterant oil—Flax, sesame, or sunflower oil; *—extra virgin olive oil, virgin olive oil or olive oil.

**Table 2:** NIRscan Nano EVM design specifications.

Parameter	Design Value
Slit ƒ-number	2.5
Slit dimensions	1.8 × 0.025 mm
DMD	DLP2010NIR
DMD ƒ-number	3.8
Spectral range	900 to 1700 nm
Spectral resolution	10 nm
Detector diameter	1 mm
Detector type	Uncooled InGaAs
Optical engine dimensions	33 × 29 × 10 mm

**Table 3:** Calibration and cross-validation statistics of developed models to quantify adulteration of olive oil with seed oils (N = 96).

Math Pre-Treatment	Parameter	N	R^2^	SEC	r^2^	SECV	RER
SG 1 2 4 4	Sesame oil	57	0.815	3.065	0.672	3.938	7.1
	Sunflower oil	39	0.886	2.718	0.653	4.958	5.6
	Flax oil	42	0.776	2.940	0.771	3.249	8.6
SG 2 2 4 4	Sesame oil	53	0.805	3.179	0.615	4.326	6.5
	Sunflower oil	38	0.852	3.014	0.608	5.041	5.6
	Flax oil	44	0.772	2.975	0.689	3.266	8.6
SG 1 2 4 4 + SNV	Sesame oil	52	0.839	2.821	0.681	4.030	6.9
	Sunflower oil	37	0.921	2.256	0.681	4.817	5.8
	Flax oil	42	0.778	2.921	0.747	3.097	9.0
SG 2 2 4 4 + SNV	Sesame oil	56	0.747	3.576	0.685	4.584	6.1
	Sunflower oil	35	0.313	6.554	0.276	6.792	4.1
	Flax oil	41	0.832	2.620	0.699	3.458	8.1
SNV + SG 1 2 4 4	Sesame oil	54	0.771	3.458	0.635	4.204	6.7
	Sunflower oil	38	0.852	3.014	0.608	5.041	5.6
	Flax oil	44	0.746	3.139	0.746	3.359	8.3
SNV + SG 2 2 4 4	Sesame oil	56	0.769	3.319	0.627	4.325	6.5
	Sunflower oil	39	0.848	3.008	0.695	5.051	5.5
	Flax oil	43	0.788	2.873	0.717	3.197	8.8

SNV—standard normal variate, N1N2N3N4—Savitzky–Golay derivative order, polynomial order of derivative, polynomial order, left, and right intervals for the derivative smoothing; R^2^—coefficient of determination for calibration, SEC—standard error of calibration, r^2^—coefficient of determination for cross-validation, SECV—standard error of cross-validation.

**Table 4:** Analytical techniques proposed to detect olive oil adulteration.

Technique	Discrimination/ Classification Procedures	Advantages	Disadvantages	Ref
Fluorescence	PCA and PLS	No sample pretreatment, Robustness to detect sunflower adulteration	Laboratory instrumentation, different models for each adulteration type	[31]
MIR	SIMCA-PLS-A	Rapid and easy-to-implement method	Lab expensive instrumentation, sometimes unstable mathematical equations	[32,33]
UV-VIS and VIS-NIR	SIMCA, SOM	Rapid analysis, easy to implement at lab level	Sample pretreatment with organic solvents for UV-VIS, Expensive instrumentation for VIS-NIR	[34,35]
NIR	PCA	No sample pretreatment, non-destructive analysis	Time-consuming for calibration development, expensive laboratory instrumentation	[36]
Raman	PCA	Non-destructive, minimum sample preparation required, rapid method	Interferences due to fluorescence properties of sample or adulterants, Expensive instrumentation, Unstable mathematical equations	[37,38]
E-nose (Voltammetric)	SPME –GC-FID or SPME-GC/MS plus PCA or PLS	High sensitivity, reproducibility, and accuracy.	Tedious sample pretreatment, expensive instrumentation, expert personnel required	[39,40]
Fast DSC	Temperature controller system, Nitrogen supply	Rapid analysis and easy-to-use technique,	Tedious and time-consuming sample pretreatment required	[41,42]
GC	Highly polar capillary column, Flame ionization or Mass Spectrometer detectors	Traditional method, accurate and precise, high reproducibility	Time-consuming sample pretreatment. expensive instrumentation, well-trained analyst	[43,44,45]
HPLC	C18 Column, Diode array, photometric of refractive index detector, high-pressure pumps	Accurate and precise, high reproducibility	Tedious and time-consuming sample pretreatment, expertise personnel required	[46,47]
NMR	NMR tube, Superconducting magnet, deuterated solvent	Short analysis time, easy sample preparation, good reproducibility	Expensive instrumentation, low sensitivity, expert personnel required	[48]
Portable NIR prototype	PCA and PLS	No sample pretreatment, on-site and real-time analysis, easy-to-use, inexpensive instrumentation	Large database for calibration development models	This work

UV—Ultraviolet, VIS—Visible, NIR—Near Infrared Spectroscopy, MIR—Medium Infrared Spectroscopy, SPME—Solid Phase Microextraction, GC—Gas Chromatography, MS—Mass spectrometry, FID—Flame Ionization Detector, DSC—differential scanning calorimetry, HPLC—High-Performance Liquid Chromatography, NMR—Nuclear Magnetic Resonance, PCA—Principal Component Analysis, PLS—Partial Least Square, SIMCA—soft independent modelling of class analogy, SOM—Self-Organizing Maps.

## Data Availability

Not applicable.
